# Mathematical Approach to Synergistic Management of Bladder Pain Syndrome/Interstitial Cystitis and Vulvodynia: A Case Series Utilizing Principal Component Analysis, Cluster Analysis, and Combination Laser Therapy

**DOI:** 10.7759/cureus.65829

**Published:** 2024-07-31

**Authors:** Nobuo Okui, Machiko A Okui

**Affiliations:** 1 Dentistry, Kanagawa Dental University, Kanagawa, JPN; 2 Urogynecology, Yokosuka Urogynecology and Urology Clinic, Kanagawa, JPN

**Keywords:** laser therapy, cluster analysis, vulvodynia, interstitial cystitis, bladder pain syndrome, synergistic management

## Abstract

This case series presents three patients with bladder pain syndrome/interstitial cystitis (BPS/IC) and vulvodynia, demonstrating the efficacy of an individualized treatment approach using cluster analysis and combination laser therapy. Principal component analysis (PCA) was used to visualize the dynamic nature of symptom clusters and guide treatment decisions. Case 1 was a 41-year-old woman initially classified as Cluster 1 (PCA coordinates: 1.65, 0.03) transitioned to Cluster 2 (-16.93, -21.75) after bladder hydrodistension. Subsequent Fotona laser (Ljubljana, Slovenia) treatment resulted in the complete resolution of symptoms. Case 2 was a 55-year-old woman, contraindicated for hormone therapy due to breast cancer history, presented as Cluster 2 (PCA coordinates: -24.16, 8.74). Fotona laser treatment shifted her to Cluster 1 (11.22, -20.22), followed by bladder hydrodistension for complete cure. Case 3 was a 49-year-old woman, initially in Cluster 0 (PCA coordinates: 1.892, 30.11), who underwent fulguration for Hunner's lesions. Posttreatment, she moved to Cluster 2 (-24.31, 1.767) and achieved full recovery after Fotona laser therapy. The dynamic nature of symptom clusters, visualized through PCA, guided treatment decisions. The PCA transformation, represented as *y* =*W^T^z*, where *z* is the standardized symptom vector and *W* is the principal component matrix, allows for the objective tracking of symptom changes. Combination Fotona laser therapy, including vaginal erbium YAG and neodymium YAG, has proven effective in managing vulvar pain, particularly when hormone therapy is contraindicated. This approach, addressing both urological and gynecological aspects, resulted in sustained symptom improvement for over 12 months in all cases. This case series highlights the synergistic relationship between BPS/IC and vulvodynia, demonstrating the efficacy of comprehensive, adaptive treatment strategies guided by mathematical analysis for complex pelvic pain syndromes.

## Introduction

Bladder pain syndrome/interstitial cystitis (BPS/IC) is a chronic condition characterized by pelvic pain and urinary symptoms, significantly impacting patients' quality of life [1,2]. The etiology of BPS/IC is multifactorial, often accompanied by various complications, such as pelvic organ inflammation, vulvodynia, and pelvic floor myofascial pain, with high reported prevalence rates of these comorbidities [3-5]. Furthermore, BPS/IC inflammation may extend to other pelvic organs, potentially contributing to the complex symptomatology of this condition [6,7].

Recent studies have emphasized the heterogeneity of BPS/IC and its frequent co-occurrence with other chronic pain conditions, particularly vulvodynia [8,9]. Machine learning approaches have identified distinct phenotypes of perceived bladder pain, revealing subgroups with varying degrees of urethral and vaginal involvement [10,11]. This deepened understanding has prompted a reevaluation of conventional treatment approaches.

Emerging research is exploring new strategies targeting both bladder and vulvar symptoms. Studies have shown promising results using topical estrogen therapy and various laser treatments to manage the symptoms of both BPS/IC and vulvodynia, suggesting a potential shared pathophysiology and the need for comprehensive, individualized approaches [12-15].

Clinically, BPS/IC symptoms often change with treatment. A key focus of recent research has been to investigate whether these changes correspond to movements between previously identified cluster groups. This dynamic perspective is crucial for the development of adaptive and personalized treatment strategies.

In this case series, we present three patients initially diagnosed with BPS/IC. By evaluating vulvar pain and applying cluster classification throughout the treatment course, we aimed to demonstrate how an individualized dynamic approach can lead to more effective treatment plans. Reflecting on the growing evidence supporting an integrated approach, we propose a novel algorithm that combines conventional BPS/IC therapies with laser treatments.

Through these case reports, we hope to contribute to an evolving understanding of the dynamic nature of BPS/IC and its relationship with vulvodynia. By sharing observations of symptom changes and treatment responses, we aim to pave the way for more nuanced, adaptive, and ultimately more effective strategies for this challenging condition.

## Case presentation

Case 1

A 41-year-old woman presented with BPS/IC symptoms. At the initial visit, the patient had a Numerical Rating Scale-11 (NRS-11) score of 8 [10,11], total Interstitial Cystitis Symptom Index (ICSI) score [10,11] of 11, total Interstitial Cystitis Problem Index (ICPI) score [10,11] of 14, Pelvic Pain and Urgency/Frequency Patient Symptom Scale (PUF) score [10,11] of 25, Overactive Bladder Questionnaire Short Form (OABq SF) score [10,11] of 26, total Overactive Bladder Symptom Score (OABSS) score [10,11] of 7, and Pelvic Floor Distress Inventory-20 (PFDI-20) score [10,11] of 42. Based on the cluster classification from a previous study [11], the patient was classified into Cluster 1. Following previous studies [8,11,13,14], the vulvodynia swab test was performed at five points: four along the hymenal scar at 2, 4, 8, and 10 o'clock positions, plus the urethral meatus. Pain intensity was recorded using a Visual Analog Scale (VAS), ranging from 0 (no pain) to 10 (maximum pain). The total score was used. The vulvodynia swab test score was 50. Table 1 shows the results of the validated questionnaires NRS-11, ICSI, ICPI, PUF, OABq SF, OABSS, and PFDI-20, the calculated cluster based on the data, and vulvodynia swab test.

**Table 1:** Clinical scores and cluster classification at different timepoints during BPS/IC treatment with vulvodynia in Case 1 NRS-11: Numerical Rating Scale-11; ICSI: Interstitial Cystitis Symptom Index; ICPI: Interstitial Cystitis Problem Index; PUF: Pelvic Pain and Urgency/Frequency Patient Symptom Scale; OABq SF: Overactive Bladder Questionnaire Short Form; OABSS: Overactive Bladder Symptom Score; PFDI-20: Pelvic Floor Distress Inventory-20; BPS/IC: Bladder Pain Syndrome/Interstitial Cystitis.

Timepoint	NRS-11	Total Vulvar Pain	ICSI Total Score	ICPI Total Score	PUF	OABq SF	OABSS Total Score	PFDI-20	Cluster
Initial visit	8	50	11	14	25	26	7	42	1
Three months after the third hydraulic distension of the bladder (T6)	8	50	6	6	12	28	4	7	2
Three months after the third Fotona Laser treatment (T12)	0	0	0	0	0	0	0	0	Cured

The patient's Body Mass Index (BMI) was 27 kg/m^2^, and she did not use hormonal contraceptives. She had no history of anxiety disorders, depression, endometriosis, fibromyalgia, gastroesophageal reflux disease (GERD), hyperlipidemia, hypertension, irritable bowel syndrome (IBS), migraine, urinary stones, polycystic ovary syndrome (PCOS), or sleep disorders.

BPS/IC was diagnosed based on cystoscopic findings and hydraulic distension of the bladder [12,16], which revealed mucosal bleeding and abrasion during distension (MBAD) and abnormal blood vessels. No Hunner's lesions were observed. Hydraulic distension of the bladder was performed according to previous literature and guidelines [12,16]. The patient was placed under general, spinal, or local anesthesia and positioned supine. A bladder catheter was inserted, and the bladder was then filled with saline to a constant pressure, typically set at 80-100 cmH_2_O, until the bladder capacity reached 200-300 ml. The bladder was maintained at this state for several minutes. Subsequently, water was drained from the bladder through a catheter. Narrow-band imaging cystoscopy was used to enhance lesion recognition.

Although vulvar pain is also important, the patient can be classified into Cluster 1. Therefore, to prioritize treating the patient's main complaint of bladder pain during storage, hydraulic distension of the bladder was performed three times at a frequency of once per month, in accordance with the guidelines [16].

**Figure 1:**
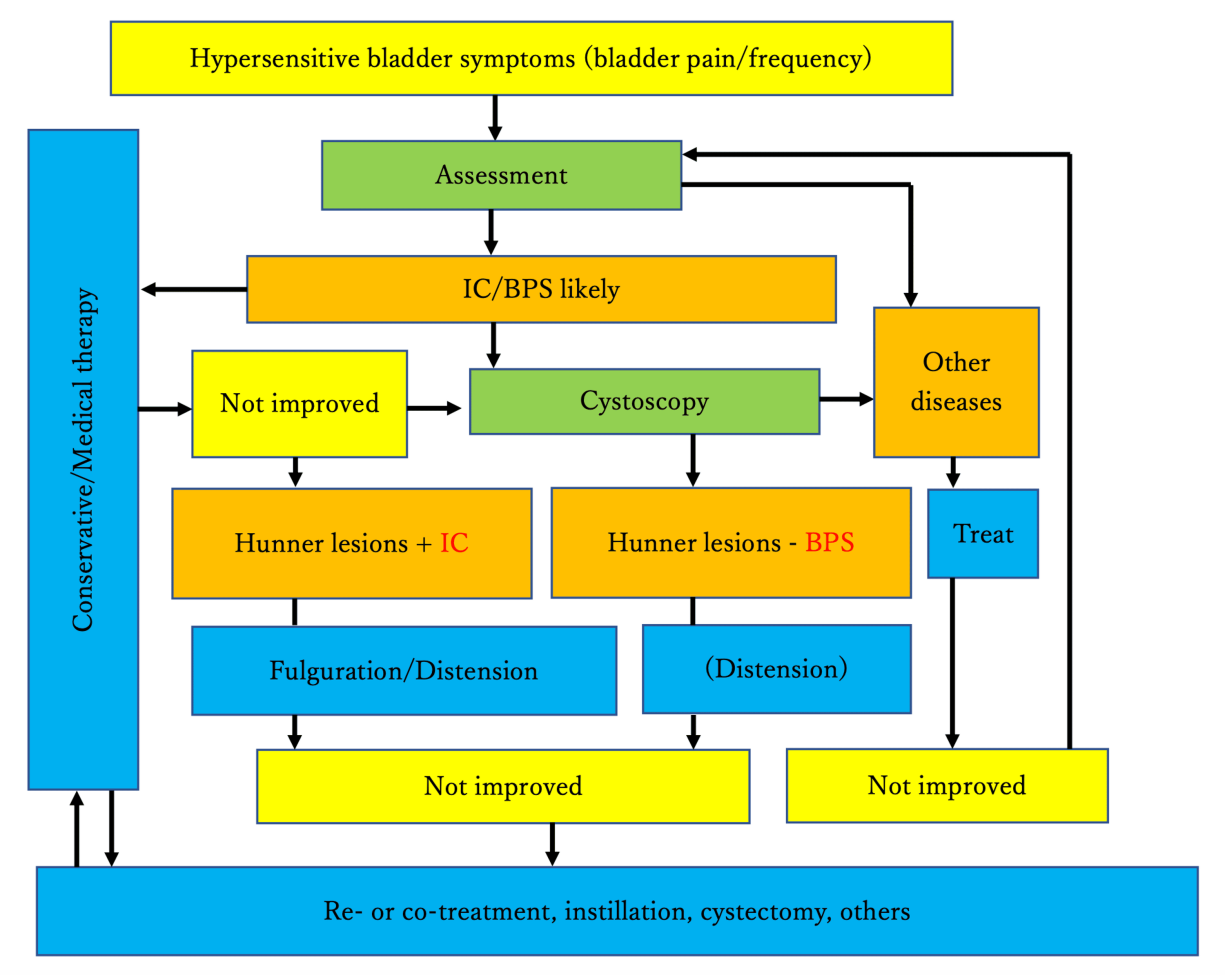
Interstitial Cystitis/Bladder Pain Syndrome (IC/BPS) according to the guidelines IC/BPS: Interstitial Cystitis/Bladder Pain Syndrome Original figure created by the authors, adapted from the concept presented in [16].

Figure 1 shows the treatment algorithm according to these guidelines [16]. While the guidelines recommend bladder hydrodistension (recommendation grade B/C), they do not specify the number of treatments or interval between them. The guidelines also state that multiple procedures may not decrease bladder capacity. However, Asian guidelines, while recommending bladder hydrodistension, note that the procedure itself carries the risk of bladder perforation. Therefore, we developed an implementation plan under the condition that it should be implemented carefully. Simultaneously, as indicated in the guidelines, we informed the patients about reports showing pain reduction through dietary therapy, including restricted intake of tomatoes, tomato products, soybeans, tofu products, spices, excessive potassium, citrus fruits, and high-acidity-inducing substances, and encouraged a review of their diet. The patient did not wish to use the medications presented in the guidelines, such as duloxetine, gabapentin, montelukast, and sildenafil. Regarding nonsteroidal anti-inflammatory drugs, the patient had been prescribed these drugs at multiple medical institutions prior to consultation, but as they had not been effective, the patient desired to completely discontinue their use.

Following the treatment pathway outlined in Figure 1, and considering the patient's preferences and risk factors, we proceeded with hydraulic distension of the bladder. Initially, this reduced the pain during bladder storage. However, it also led to intensification of pre-existing pain before and after urination and while sitting, which varied in intensity. This pain was felt in the perineum, labia, and viscera and became very distressing for the patient.

Three months after the third hydraulic distension of the bladder (T6), a re-evaluation was conducted, and the following changes in symptoms were observed, confirming that the cluster had shifted from 0 to 2. At this point, local female hormone administration was performed for three months in the vulvovaginal area, but the pain did not improve. Therefore, the Fotona laser treatment [12-14] was performed (Fotona d.o.o., Ljubljana, Slovenia). This is a combination treatment of vaginal non-ablative erbium YAG laser (VEL) and neodymium YAG laser (NdYAG) [12-14]. After cleansing the perineum, laser irradiation was performed using a handpiece connected to the SP Dynamis (Fotona). The VEL protocol used a wavelength of 2,940 nm. This method consisted of two steps. Step 1 involved VEL with an angular adapter and PS03 handpiece at 10.00 J/cm², 2.0 Hz, and 7 mm spot size, applied in six segments three times to the vaginal anterior wall. Step 2 uses VEL with a circular adapter and R11 handpiece at 3.00 J/cm², 2.0 Hz, and 7 mm spot sizes, covering the entire vaginal wall in a 360-degree circular pattern from the cervix toward the introitus.

**Figure 2:**
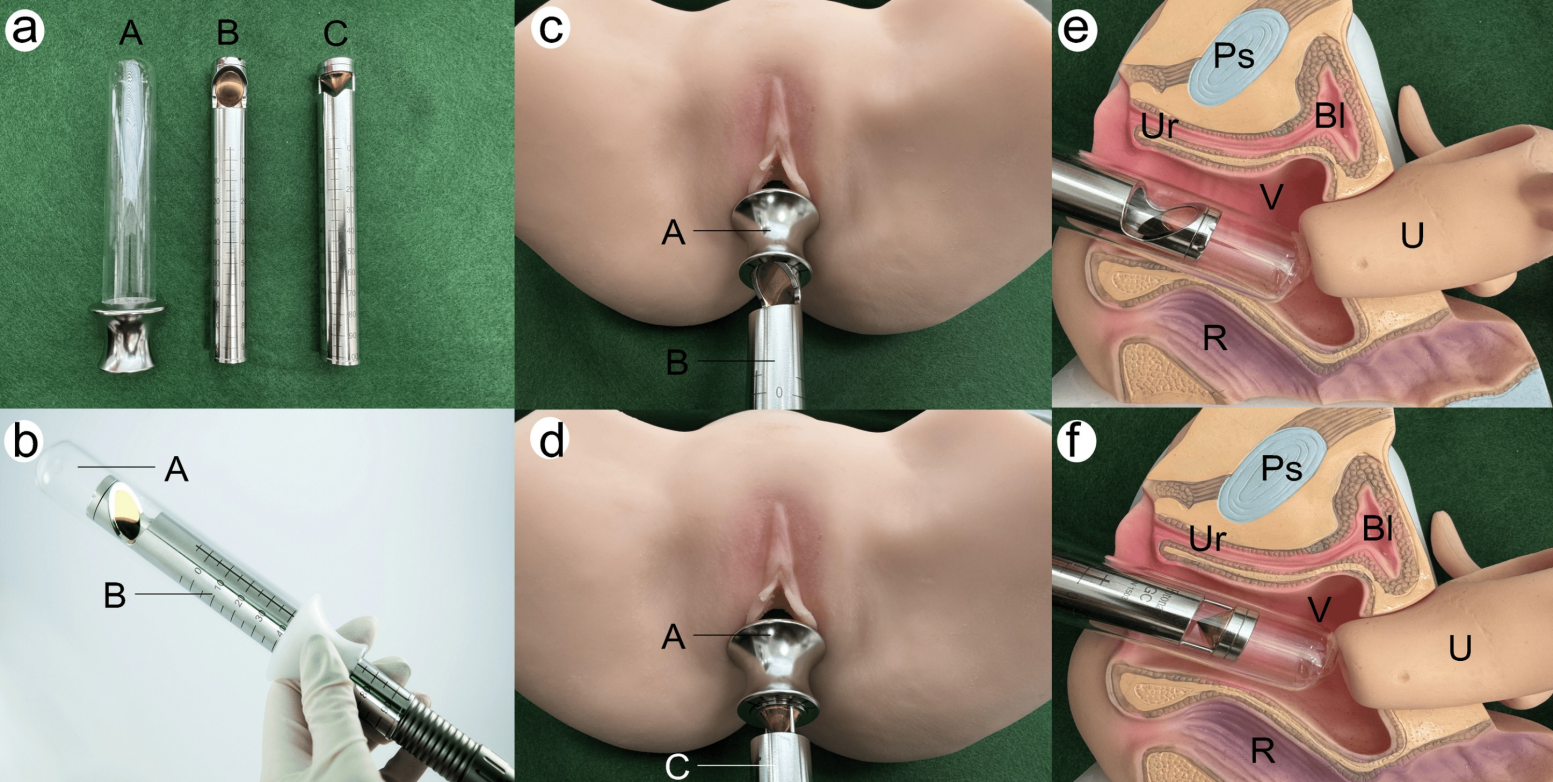
Vaginal non-ablative erbium YAG laser (VEL) tools and anatomical models a. Tools used for VEL: A, glass speculum; B, PS03-GA handpiece for irradiating the anterior vaginal wall; C, R11-GC handpiece for irradiating the entire vaginal circumference. b. Insertion of PS03-GA (B) into the glass speculum (A). c. Insertion of PS03-GA (B) handpiece into a silicone model after inserting the glass speculum (A). The photos in this figure are all original images. Additionally, the photographs of the equipment used were taken with permission from Fotona d.o.o (Ljubljana, Slovenia). d. Insertion of R11-GC (C) handpiece into the silicone model after inserting the glass speculum (A). e. Glass speculum (A) and handpiece PS03-GA (B) inserted in a plastic anatomical model, showing the anatomical position. f. Glass speculum (A) and handpiece R11-GC (C) inserted in a plastic anatomical model, showing the anatomical position. Ps: pubic symphysis; Ur: urethra; V: vagina; R: rectum; Bl: bladder; U: uterus.

Figure 2 shows the tools and models used to understand VEL. Figure 2A shows the tools used for VEL, including a glass speculum (A) inserted into the vagina, handpiece PS03-GA (B) for irradiating only the anterior vaginal wall, and handpiece R11-GC1 (C) for irradiating the entire vaginal circumference. Figure 2B shows the insertion of PS03-GA (B) into a glass speculum (A). Figure 2C shows the insertion of the PS03-GA (B) handpiece into a silicone model after inserting the glass speculum (A), washing the patient's vagina with a disinfectant, drying it with a cotton swab, and applying local anesthesia to the surface before treatment. Figure 2D shows the insertion of the R11-GC1 (C) handpiece into the silicone model after insertion of the glass speculum (A). Figure 2E shows how the glass speculum (A) and handpiece PS03-GA (B) were anatomically inserted using a plastic anatomical model. Figure 2F shows how the glass speculum (A) and handpiece R11-GC1 (C) were anatomically inserted using a plastic anatomical model.

Immediately after the VEL treatment, Nd:YAG treatment was performed without a break. The 1064 nm Nd:YAG laser treatment was administered in a continuous PIANO pulse mode for a duration of 30 min, utilizing a non-contact R33 handpiece set at an energy density of 90 J/cm², with a five-second pulse duration and a 9 mm spot size, targeting the perineum and external genitalia.

**Figure 3:**
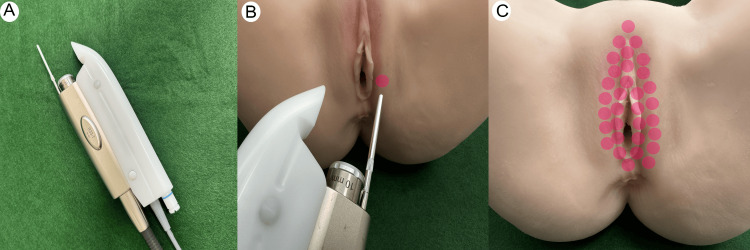
Tools and models for understanding Neodymium YAG laser (Nd:YAG) Figure 3a: Laser tools. R33 non-contact handpiece Figure 3b: Irradiation process using R33. Figure 3c: the irradiation sites The photos in this figure are all original images. Additionally, the photographs of the equipment used were taken with permission from Fotona d.o.o. (Ljubljana, Slovenia).

Figure 3 shows the tools and models used to understand the Nd:YAG. Figure 3A shows the R33 non-contact handpiece used for the Nd:YAG laser (Fotona SP Dynamis, PIANO mode, spot size of 9 mm, PIANO pulse mode (5 s), and fluence of 90 J/cm^2^). Figure 3B shows the irradiation process using an R33 (D) handpiece on the silicone model. The red circles represent the irradiation images. Figure 3C indicates the irradiation sites.

Fotona laser treatment was administered once a month, for a total of three sessions. Patients were advised to avoid sexual intercourse for one week after each session. The evaluation conducted three months after the third Fotona laser treatment (T12) showed improvement in all pain metrics, indicating successful treatment. Furthermore, no symptom recurrence was observed in the subsequent 12 months.

**Figure 4:**
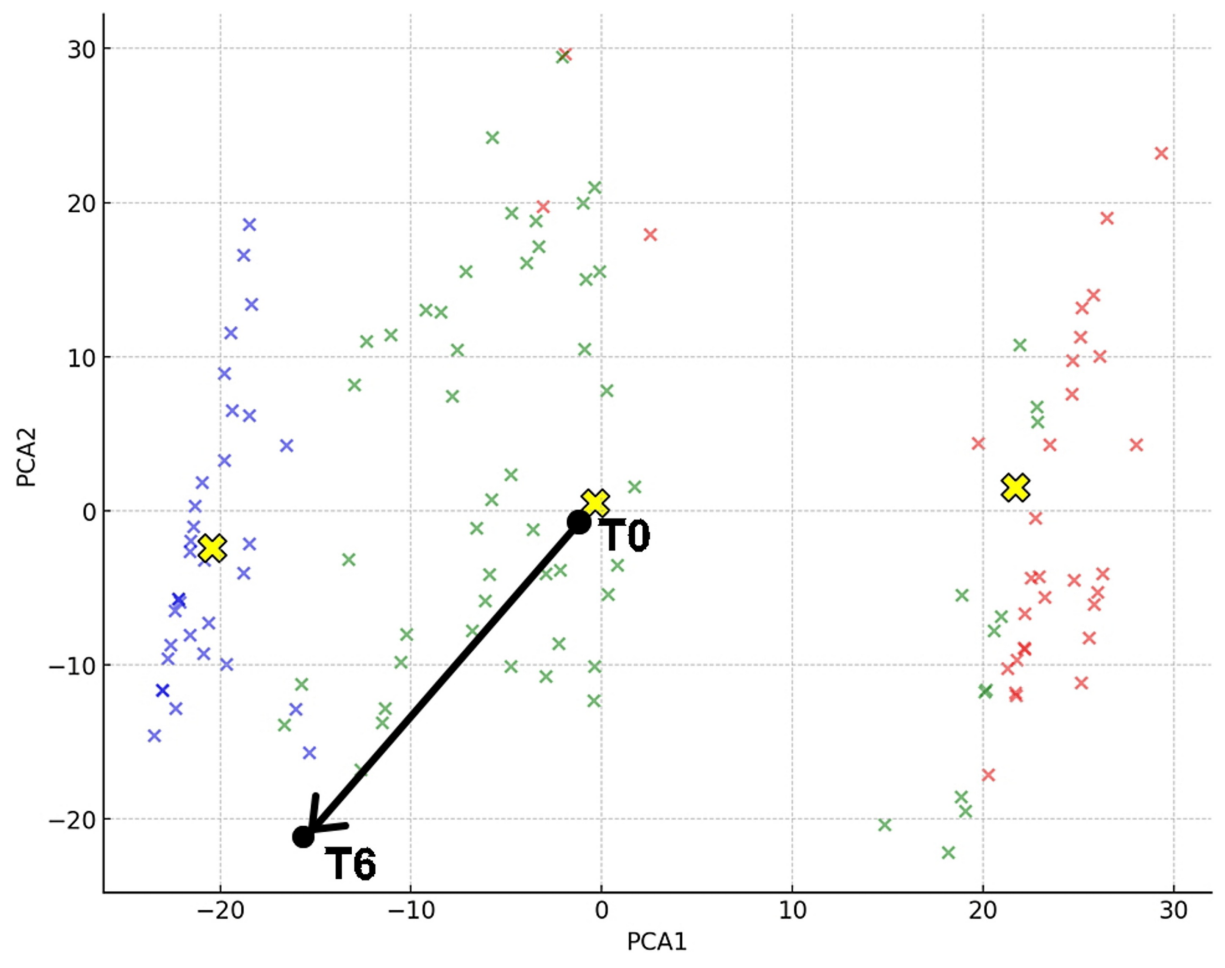
Three Clusters and Changes in Coordinates for Case 1 The two black dots represent Case 1 at the initial visit (T0) and three months after bladder hydrodistension treatment (T6). PCA1 and PCA2 are the principal components that capture the maximum variance in the data, visually distinguishing the symptom profiles of each cluster. Cluster 0 is represented in red, Cluster 1 in green, and Cluster 2 in blue [11]. The y-axis represents PCA2, and the x-axis represents PCA1. The yellow “X” marks indicate the centroids of the clusters, capturing the central points of each cluster in the reduced dimensional space [11]. PCA, principal component analysis

Figure 4 shows that at the initial visit (T0), the patient was classified as Cluster 1, and at T6 as Cluster 2. New data points were inserted into the graph representing the three clusters of BPS/IC patients derived from standardized questionnaires in previous research. The coordinates were calculated using Python code by implementing principal component analysis (PCA). PCA is a technique that transforms high-dimensional data into lower-dimensional data by finding new coordinate axes that maximize the variance of the data. As a result, the coordinates of the initial visit (T0) and three months after hydraulic distension (T6) were obtained and are shown in Figure 4. The coordinates for T0 were (PCA1, PCA2) = (1.65, 0.03), and those for T6 were (−16.93, −21.75).

This process can be mathematically represented for the standardization and PCA transformation of new data points. The given features were defined as follows: Age (X_1_), NRS-11 (X_2_​), ICSI Total (X_3_​), ICPI Total (X_4_​), PUF (X_5_​), OABq SF (X_6_​), OABSS Total (X_7​_), and PFDI20 (X_8​_).

Each feature is standardized using the formula zi=Xi−μiσi, where z=(z1,z2,z3,z4,z5,z6,z7,z8).

The standardized data points *z* are then projected into the principal component space using the formula *y*=*W^T^z*, where *W* is the matrix of principal component vectors. The transformed data points *y* are calculated as *y*=(y1,y2)=(PCA1,PCA2).

In this way, the coordinates of the new data points in the principal component space are obtained. This method allows the integration of new patient data into the existing PCA plot. By following these steps, it is possible to dynamically update the PCA plot with new data, ensuring that the new points are accurately represented in the context of the existing principal components. This approach facilitates a more comprehensive and adaptive analysis of patient data, enhancing the understanding and management of complex conditions such as BPS/IC.

Case 2

A 55-year-old woman presented to the clinic with BPS/IC symptoms. At the age of 43, she underwent tumor resection for right breast cancer. Although the patient did not receive hormone therapy or chemotherapy, female hormone therapy was contraindicated. At the initial visit, the patient had an NRS-11 score of 9, total vulvar pain of 50, total ICSI score of 6, total ICPI score of 10, PUF score of 16, OABq SF score of 18, total OABSS score of 3, and PFDI-20 score of 7. Based on the cluster classification, the patient was classified as Cluster 1. The patient's body mass index was 20.7, and she did not use hormonal contraceptives. She had no history of anxiety disorders, depression, endometriosis, fibromyalgia, GERD, hyperlipidemia, hypertension, IBS, migraine, urinary stones, PCOS, or sleep disorders.

During the medical interview, the patient reported bladder pain when the bladder was full, but there was severe pain in the urethra, making endoscopic examination impossible. At this point, it was predicted that forcibly dilating the urethra would likely result in uncontrolled urethral pain. However, because the patient's main complaint was vaginal pain, priority was given to the treatment of vulvar pain. Fotona laser treatment (VEL + NdYAG) was performed on the vulvovaginal area once a month for a total of three sessions. As a result, the symptoms improved three months after the third Fotona laser treatment (T6). Table 2 shows the progress of treatment.

**Table 2:** Clinical scores and cluster classification at different timepoints during BPS/IC treatment with vulvodynia in Case 2 NRS-11: Numerical Rating Scale-11; ICSI: Interstitial Cystitis Symptom Index; ICPI: Interstitial Cystitis Problem Index; PUF: Pelvic Pain and Urgency/Frequency Patient Symptom Scale; OABq SF: Overactive Bladder Questionnaire Short Form; OABSS: Overactive Bladder Symptom Score; PFDI-20: Pelvic Floor Distress Inventory-20; BPS/IC: Bladder Pain Syndrome/Interstitial Cystitis.

Time Point	NRS-11	Vulvodynia Total	ICSI Total	ICPI Total	PUF	OABq SF	OABSS Total	PFDI20	Cluster
Initial Visit	9	50	6	10	16	18	3	7	2
Three Months After Third Fotona Laser Treatment (T6)	4	0	4	4	10	12	3	20	1
Three Months After Bladder Hydrodistension (T10)	0	0	0	0	0	0	0	0	Cured

After Fotona laser treatment, the pain in the vulvovaginal area was significantly reduced, but the patient complained that the pain during bladder filling persisted.

**Figure 5:**
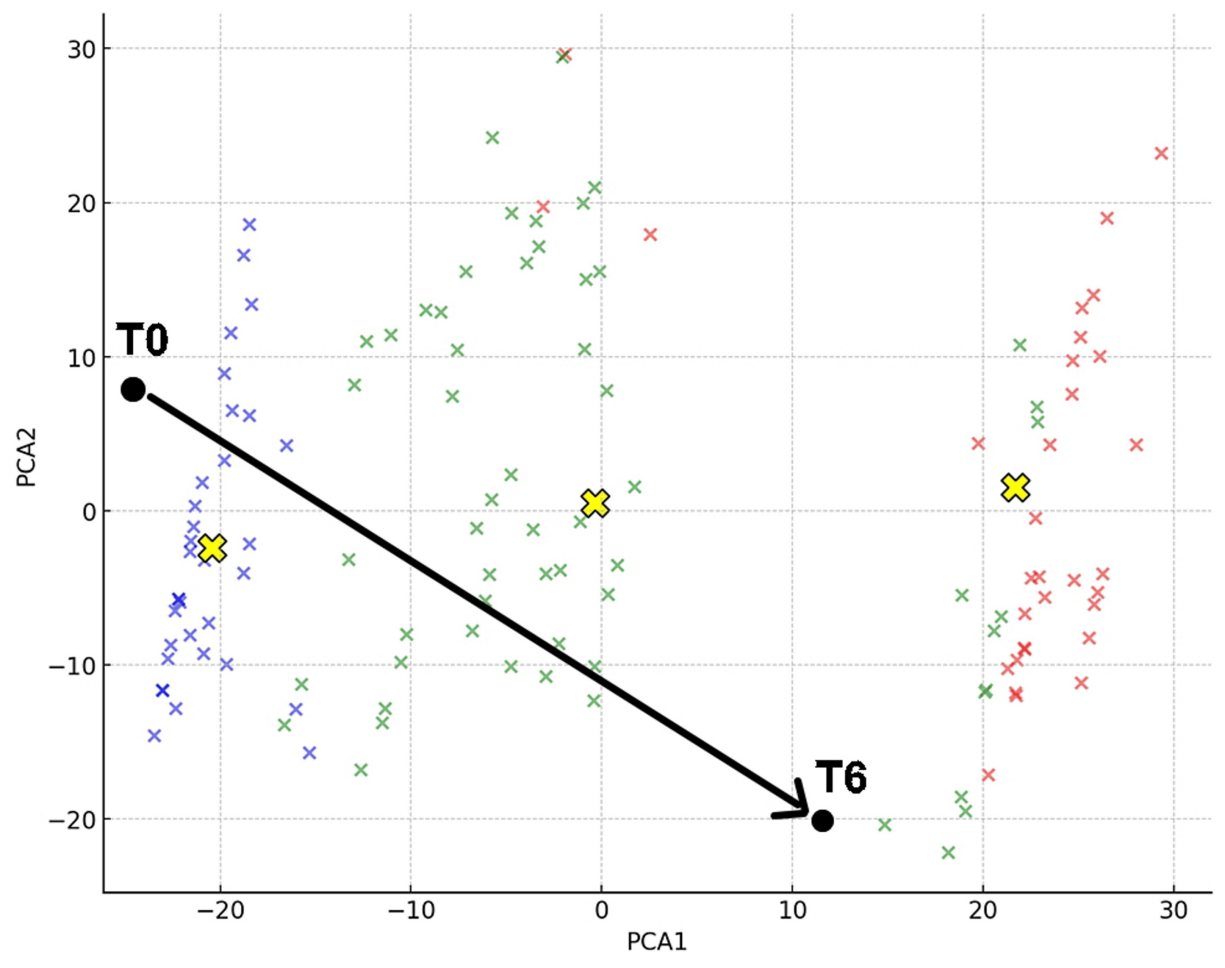
Three Clusters and Changes in Coordinates for Case 2 The two black dots represent Case 1 at the initial visit (T0) and three months after Fotona laser (Fotona d.o.o., Ljubljana, Slovenia) treatment (T6). PCA1 and PCA2 are the principal components that capture the maximum variance in the data, visually distinguishing the symptom profiles of each cluster. Cluster 0 is represented in red, Cluster 1 in green, and Cluster 2 in blue [11]. The y-axis represents PCA2, and the x-axis represents PCA1. The yellow “X” marks indicate the centroids of the clusters, capturing the central points of each cluster in the reduced dimensional space [11]. PCA, principal component analysis

As urethral pain had also been alleviated due to the effects of laser treatment, following the guidelines [16] shown in Figure 1, cystoscopy was performed to assess Hunner's lesions and to diagnose BPS/IC. Endoscopic findings revealed an MBAD during hydraulic distension of the bladder. Hunner's lesions were not observed, and the patient was diagnosed with BPS/IC. Three months later, evaluation showed that pain during bladder storage was also significantly reduced (T10). Pain improved in all items, indicating that the treatment was successful. No recurrence was observed 12 months after the completion of treatment. Figure 5 shows that in the cluster classification, the initial visit (T0) was Cluster 2, and T6 was Cluster 1. The coordinates at T0 were (PCA1, PCA2) = (-24.16, 8.74). At T6, they were (11.22, -20.22).

Case 3

A 49-year-old woman presented to the clinic with BPS/IC symptoms. At the initial visit, the patient had an NRS-11 score of 8, total vulvar pain of 42.5, total ICSI score of 13, total ICPI score of 12, PUF score of 25, OABq SF score of 25, total OABSS score of 7, and a PFDI-20 score of 42. Based on the cluster classification, the patient was classified as Cluster 0. The patient's BMI was 20.1, and she was not using hormonal medications. She had no history of anxiety disorder, depression, endometriosis, fibromyalgia, GERD, hyperlipidemia, hypertension, IBS, migraine, urinary stones, PCOS, or sleep disorders.

The patient's main complaint was bladder storage pain. Ultrasound findings revealed no particular issues, except for a uterine myoma measuring approximately 1 cm. In accordance with the guidelines [16] shown in Figure 1, cystoscopy was performed to assess for Hunner's lesions and diagnose BPS/IC. Endoscopic findings revealed an MBAD and abnormal blood vessels during hydraulic distension of the bladder, and one Hunner's lesion was confirmed. BPS/IC was diagnosed, and vulvodynia was observed; however, based on the cluster classification, she was considered Cluster 0. Following the treatment pathway outlined [16] in Figure 1, which recommends fulguration/distension for BPS/IC patients with Hunner's lesions, endoscopic fulguration of the Hunner's lesion was performed to prioritize the treatment of pain during bladder storage. The pain significantly improved after this intervention. Hydraulic distension of the bladder was performed simultaneously.

However, pain in the perineum, labia, and viscera began to be felt while sitting, and re-evaluation was conducted three months after the first hydraulic distension of the bladder (T4). Table 3 shows the progress of treatment.

**Table 3:** Clinical scores and cluster classification at different timepoints during BPS/IC treatment with vulvodynia in Case 3 NRS-11: Numerical Rating Scale-11; ICSI: Interstitial Cystitis Symptom Index; ICPI: Interstitial Cystitis Problem Index; PUF: Pelvic Pain and Urgency/Frequency Patient Symptom Scale; OABq SF: Overactive Bladder Questionnaire Short Form; OABSS: Overactive Bladder Symptom Score; PFDI-20: Pelvic Floor Distress Inventory-20; BPS/IC: Bladder Pain Syndrome/Interstitial Cystitis.

Timepoint	NRS-11	Total Vulvar Pain	ICSI Total Score	ICPI Total Score	PUF	OABq SF	OABSS Total Score	PFDI20	Cluster
Initial Visit	8	42.5	13	12	25	25	7	42	1
Three Months After First Bladder Hydrodistension (T4)	5	46.25	4	3	7	8	0	7	2
Three Months After Third Fotona Laser Treatment (T10)	0	0	0	0	0	0	0	0	Cured

**Figure 6:**
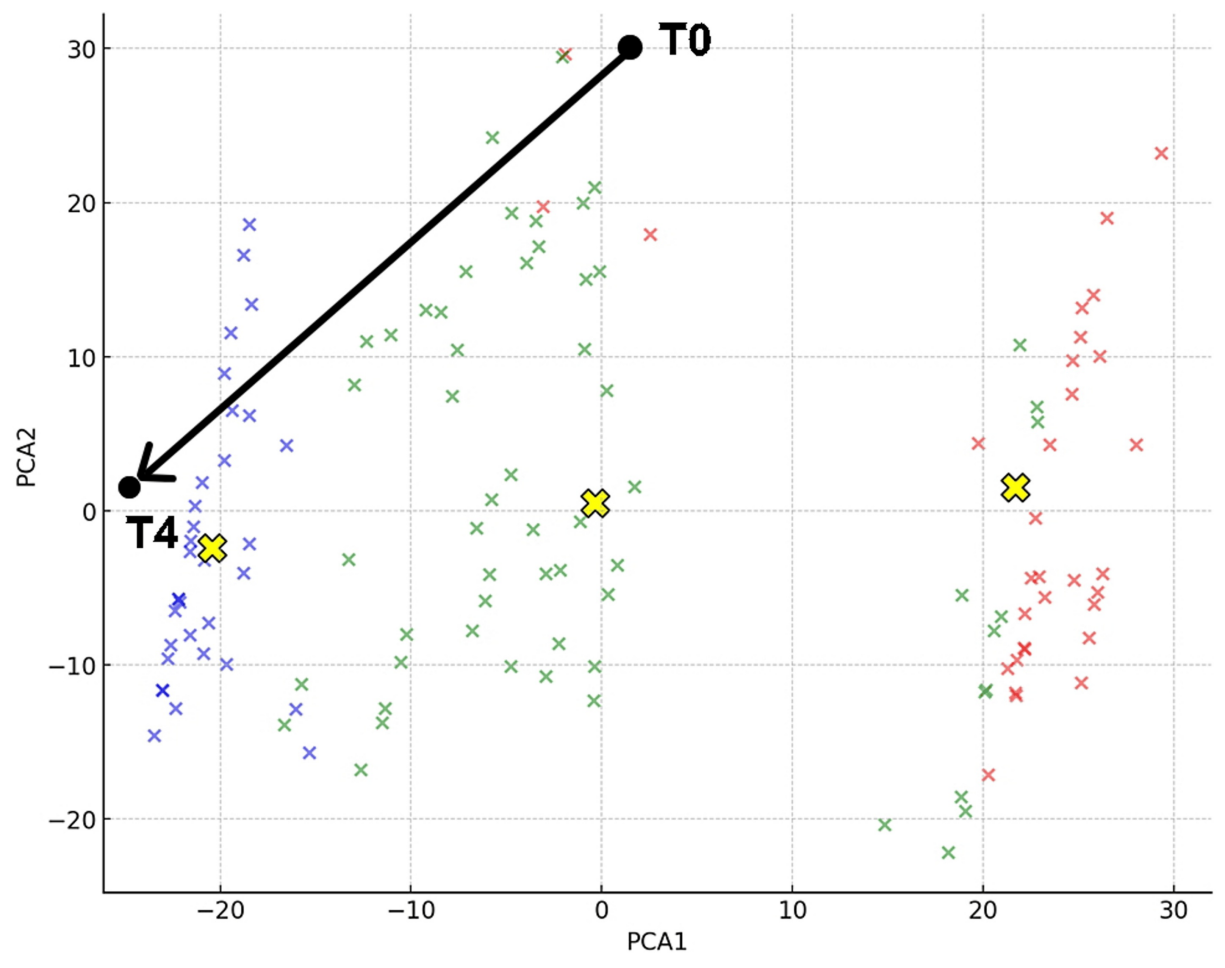
Three Clusters and Changes in Coordinates for Case 3 The two black dots represent Case 1 at the initial visit (T0) and three months after Fotona laser (Fotona d.o.o., Ljubljana, Slovenia) treatment (T6). PCA1 and PCA2 are the principal components that capture the maximum variance in the data, visually distinguishing the symptom profiles of each cluster. Cluster 0 is represented in red, Cluster 1 in green, and Cluster 2 in blue [11]. The y-axis represents PCA2, and the x-axis represents PCA1. The yellow “X” marks indicate the centroids of the clusters, capturing the central points of each cluster in the reduced dimensional space [11]. PCA, principal component analysis

Based on this data, it was considered that the pain at the initial visit was triggered by the Hunner's lesion, but it was judged that the treatment of vulvodynia should be prioritized for the remaining pain. At this point, it was confirmed that she had moved to Cluster 2. The patient had previously experienced symptoms of thrombosis when using hormone replacement therapy and did not wish to use it again. Therefore, Fotona laser treatment (VEL + NdYAG) was performed on the vulvovaginal area once a month for a total of three sessions. As a result, the evaluation three months after the third Fotona laser treatment (T10) showed that pain improved in all items, indicating that the treatment was successful. No recurrence was observed for 12 months thereafter. Figure 6 shows that, in the cluster classification, the initial visit (T0) was Cluster 2, and T4 was Cluster 1. The coordinates at T0 were (PCA1, PCA2) = (1.892, 30.11). At T4, they were (-24.31, 1.767).

## Discussion

This case series demonstrates the importance of an individualized approach in the treatment of BPS/IC, with a particular focus on its association with vulvodynia and the efficacy of novel treatment modalities [1,2].

The relationship between BPS/IC and vulvodynia, as reported by Gardella et al. showing a high prevalence of vulvar pain in BPS/IC patients, was confirmed in our case series [9]. Specifically, as demonstrated in the UNICORN-1 study, BPS/IC patients exhibit significant deterioration in vaginal health and sexual function, highlighting the importance of comprehensively evaluating these symptoms [8]. Moreover, the identification of distinct bladder pain phenotypes through machine learning approaches, as shown in studies by Mwesigwa et al. and Okui et al., supports the utility of cluster classification [10,11]. Additionally, it has been confirmed that BPS/IC symptoms fluctuate over time [17].

Our case series illustrates the usefulness of cluster classification for understanding the relationship between BPS/IC and vulvodynia. In Cases 1 and 3, patients initially classified as Cluster 1 transitioned to Cluster 2 over the course of treatment. In Case 2, the patient moved from Cluster 2 to Cluster 1. This finding demonstrates that BPS/IC symptoms change dynamically, necessitating adaptive treatment strategies. The use of cluster classification allows for objective evaluation of symptom changes, leading to appropriate treatment choices [11].

The effectiveness of the PCA and clustering approaches was mathematically validated. The PCA dimensionality reduction is represented by the equation:

$X=U\Sigma V^{T}$

where X is the original data matrix, U and V are singular vectors, and Σ is the singular value matrix. This transformation enables the visualization of high-dimensional symptom data in two dimensions, where $X\in \mathbb{R}^{n \times p}$ is the original data matrix of n patients and p symptoms, $U\in \mathbb{R}^{n \times n}$ and $V\in \mathbb{R}^{p \times p}$ are orthogonal matrices, and $\Sigma \in \mathbb{R}^{n \times p}$ is a rectangular diagonal matrix of singular values. This transformation enables the visualization of high-dimensional symptom data in two dimensions.

To quantify inter-cluster movement, Euclidean distance was used:

$\small d=\sqrt{(x_{2}-x_{1})^{2}-(y_{2}-y_{1})^{2}}$

where (x₁, y₁) and (x₂, y₂) are the PCA coordinates before and after the treatment, respectively. This distance metric allows for the numerical assessment of symptom changes and objective evaluation of treatment efficacy.

Future research could incorporate nonlinear dimensionality reduction techniques such as t-distributed Stochastic Neighbor Embedding (t-SNE):

$p_{ij} = \frac{\exp(-\|x_i - x_j\|^2 / 2\sigma_i^2)}{\Sigma_{k \neq i} \exp(-\|x_i - x_k\|^2 / 2\sigma_i^2)}$

where $p_{ij}$ is the similarity between data points $x_i$ and $x_j$, and $\sigma_i$ is the variance of the Gaussian centered at $x_i$.

Additionally, dynamic clustering algorithms considering time-series data could be explored, potentially enabling more precise modeling of the interaction between BPS/IC and vulvodynia. For instance, a time-dependent Gaussian Mixture Model could be employed:

$p(x_t \mid z_t = k) = N(x_t \mid \mu_k(t), \Sigma_k(t))$

where $z_t$ is the cluster assignment at time $t$, and $\mu_k(t)$ and $\Sigma_k(t)$ are time-dependent mean and covariance for cluster $k$. These advanced techniques could potentially reveal complex, non-linear relationships in the symptom data and capture the dynamic nature of BPS/IC and vulvodynia over time.

Regarding the efficacy of laser therapy, the UNICORN-2 study reported the effectiveness of VEL treatment [12]. Furthermore, Okui et al. advanced the treatment to VEL + Nd:YAG combination therapy, enhancing its therapeutic effects [13]. The long-term safety of this treatment approach is also noteworthy [17].

In our case report, VEL + Nd:YAG showed high efficacy, particularly for vulvar pain. In Case 2, in which hormone therapy was contraindicated due to a history of breast cancer, laser treatment provided safe and effective symptom improvement. This suggests the potential of laser therapy as an alternative to hormone therapy [13,15,18,19]. The absence of recurrence in all cases over a 12-month period demonstrated the sustained effect of this treatment approach. Moreover, as reported in the UNICORN-3 study, the side effects of laser treatment were minimal and temporary, confirming its high safety profile [14].

As a comprehensive treatment approach, the UNICORN-3 study demonstrated that combination laser therapy targeting both the vagina and vulva is effective for both BPS/IC and vulvodynia [14]. This calls for an approach that views BPS/IC not merely as a urological condition but from both urological and gynecological perspectives [4,8,9]. In our case series, combining direct approaches to the bladder with vulvar approaches resulted in significant symptom improvement, supporting the concept that BPS/IC and vulvodynia are synergistic syndromes [9,14].

Building on our findings and emerging trends in personalized medicine, future research should focus on validating and refining our dynamic clustering approach. Wang et al. demonstrated the potential of personalized predictive models based on patient similarity to improve the diagnostic accuracy [20]. Similarly, in BPS/IC research, comparing the performance of clustering-based personalized models with traditional models using the entire patient dataset can further establish the effectiveness of our approach. Future studies should aim to develop more sophisticated machine-learning algorithms that can continuously update patient clusters as treatment progresses, integrating real-time symptom data and treatment responses. This could lead to highly adaptive treatment strategies that evolve with the changing symptom profile of the patient. Additionally, larger-scale studies incorporating diverse patient populations are needed to validate the generalizability of our findings and to identify potential subgroups that may benefit the most from specific treatment modalities.

## Conclusions

This case series demonstrates the efficacy of an individualized, dynamic approach to treating BPS/IC and vulvodynia using cluster analysis and combination laser therapy. The synergistic management strategy, incorporating both bladder-focused treatments and vulvar laser therapy, led to significant symptom improvement and sustained relief in all cases. Future research should focus on larger-scale studies to validate this approach and explore its potential for improving quality of life in patients with complex pelvic pain syndromes.
